# Previously Unidentified Gene Variation Associated with Fabry Disease: The Impact on One Family

**DOI:** 10.1155/2020/8899703

**Published:** 2020-09-19

**Authors:** Amaresh R. Vanga, Samantha A. Schrier Vergano, Jolanta Kowalewska, Thomas R. McCune

**Affiliations:** ^1^Division of Nephrology, Department of Internal Medicine, Eastern Virginia Medical School, Norfolk, VA, USA; ^2^Division of Medical Genetics and Metabolism, Children's Hospital of the King's Daughters, Norfolk, VA, USA; ^3^Department of Pediatrics, Eastern Virginia Medical School, Norfolk, VA, USA; ^4^Department of Pathology and Anatomy, Eastern Virginia Medical School, Norfolk, VA, USA; ^5^Center for Congenital Kidney Diseases, Eastern Virginia Medical School, Norfolk, VA, USA

## Abstract

Fabry disease is an X-linked lysosomal storage genetic disorder associated with over 1000 mutations in the *alpha-galactosidase-A* gene region. We report here a 69-year-old male who underwent a kidney biopsy to evaluate progressive renal failure. He was found to have zebra bodies in visceral epithelial cells on biopsy, with electron microscopy showing inclusions within the cytoplasm of multiple podocytes consistent with Fabry disease. An alpha-galactosidase level was found to be 21 nm/hr/mg (normal range 50–150 nm/hr/mg). Genetic studies revealed a missense variant in the *GLA* gene with alanine replaced by cysteine at position 682 (c.682 A > C, p.N228H) that had not been previously associated with Fabry disease. The same variant was detected in two additional family members. The pathologic findings, clinical features, and low alpha-galactosidase level suggest that the c.682 A > C variant is associated with Fabry disease.

## 1. Introduction

Fabry disease is an X-linked lysosomal storage disease caused by a deficiency in the alpha-galactosidase enzyme, which affects both males and females. The signs and symptoms of Fabry disease include skin angiokeratomas, neuropathic limb pain, abdominal complaints, corneal opacities, transient ischemic events/strokes, left ventricular hypertrophy, and renal impairment. These can be manifestations and complications of other diseases such as diabetes and hypertension; therefore, Fabry disease may not be considered. Screening of populations at risk for Fabry disease based on ESRD [1–4], cryptogenic strokes [5], and left ventricular hypertrophy [6] found an instance of 1.0% or less. There are over 1,000 known mutations associated with the region that codes for the alpha-galactosidase enzyme [7]. Because of the large number of gene variations, there is a great deal of heterogeneity in the presentation of this multiorgan disease. This article presents a case of Fabry disease due to the *GLA* pathogenic variant not previously associated with the disease.

## 2. Case Report

**Figure 1:**
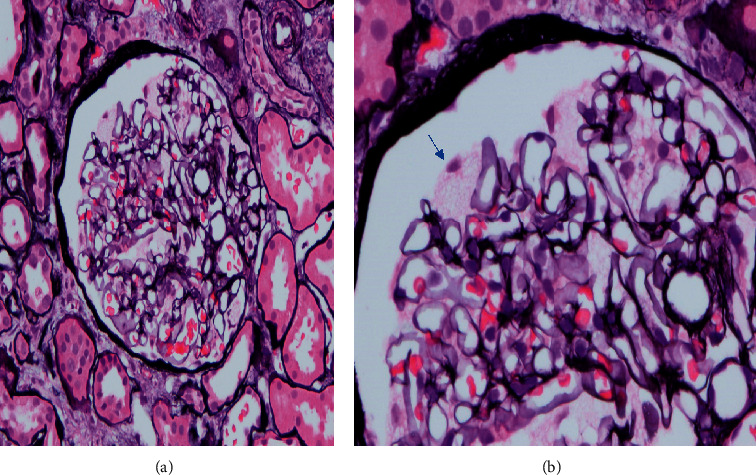
Normocellular glomerulus showing podocytes with foamy cytoplasm (blue arrow). Hematoxylin eosin stain; original magnification 200x (a) and 400x (b).

**Figure 2:**
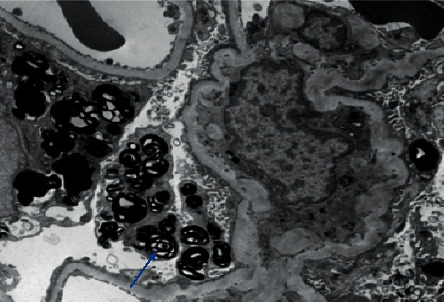
Electron microscopy showing podocytes containing dark lamellated osmophilic inclusions, some with appearance of zebra bodies (blue arrow). Transmission electron microscopy; original magnification 3000x.

A 69-year-old male with a past medical history of hypertension, diabetes, morbid obesity requiring gastric bypass, and metastatic prostate cancer presented for evaluation of progressive kidney disease with subnephrotic-range proteinuria. His glomerular filtration declined by greater than 5 ml per minute per 1.73 m^2^ per year. His diabetes mellitus was diet controlled. Medications included atorvastatin, amlodipine, aspirin, calcium carbonate, and leuprolide injection every three months. There were no known drug allergies. The family history was positive for renal failure of undetermined etiology in a brother in his forties. His review of systems was remarkable for peripheral neuropathy with unstable balance, but he denied any symptoms of hypohidrosis, extremity pain, GI complaints, rashes, or cardiac issues. He denied any skin issues. Physical exam revealed a blood pressure of 160/78 mmHg, pulse of 72, and BMI of 24.5. There were no abnormal physical findings of his lungs, heart, abdomen, extremities, or skin. The serum laboratory revealed a creatinine of 4.3 mg/dl and BUN of 47 mg/dl and CO_2_ of 19 mg/dl. The remainder of the electrolytes was within normal limits. On CBC, there was hemoglobin of 10.9 but normal platelets and WBC. Urinalysis was remarkable for mild proteinuria. The urine protein/creatinine ratio was 2.2. Initial evaluation of SPEP, UPEP, and free light chains failed to demonstrate any abnormalities. Ultrasound of kidneys showed slightly atrophic kidneys with echogenic parenchyma. A kidney biopsy was performed to complete the evaluation. Light Microscopic evaluation showed normocellular glomeruli with uniform capillary walls and prominent visceral epithelial cells (podocytes) with foamy cytoplasm (Figure 1) and myelin/zebra bodies in visceral epithelial cells (Figure 2) suggesting Fabry disease. Ultrastructural examination revealed osmophilic lamellated inclusions within the cytoplasm of multiple podocytes with concentric of striped appearance (zebra bodies) suggestive of defective lysosomal metabolism of glycolipids, as seen in Fabry disease.

After the biopsy report has been explained to the patient, his brother confirmed that he had been told he had Fabry disease. Lysosomal GL-3 was not drawn as biopsy findings and clinical symptomology were consistent with this diagnosis. The family history is limited as his brother is estranged. He had not had any genetic testing.

The alpha-galactosidase level was 21 nm/hr/mg protein (50–150 nm/hr/mg protein). Genetic studies were sent to EGL fenetics for evaluation. They detected a missense variant in the *GLA* gene sequence in which the nucleotide alanine is replaced by cysteine at position 682 in (c.682 A > C). The variation c.682 A > C in the GLA sequence was a known variant but previously had not been associated with clinical Fabry disease. Genetic testing performed on his daughter and his grandson confirmed that they were positive for this same variant. The testing of other family members is ongoing. Testing of this mutation by Cambridge Biomedical determined that Migalastat chaperon therapy would be appropriate.

## 3. Discussion

This case suggests that the c.682 A > C variant is associated with Fabry disease based on low alpha-galactosidase levels and pathologic lesions on kidney biopsy. The late age at onset of renal disease and only a 50% decrease in alpha-galactosidase level suggest an atypical variant. However, the fact that the patient's brother developed ESRD in his forties suggests a more classic form of the disease. Another Fabry disease mutation, p.R301Q, which is not related to this mutation, is also associated with both classic and atypical presentations [8]. This suggests that both classic and atypical presentations can be observed in multiple Fabry disease mutations.

The diagnosis of Fabry disease requires a high index of suspicion as many symptoms, such as hypohidrosis, go unrecognized. Other Fabry disease symptoms related to the gastrointestinal, cardiac, and neurologic systems may be attributed to other common conditions such as diabetes mellitus and hypertension. This patient had multiple indicators of Fabry disease, such as a male relative with ESRD at an early age and neuropathy not in proportion to his diet-controlled diabetes yet went undiagnosed. Measuring serum alpha-galactosidase enzyme levels can be a useful screening study in male patients but can yield false-positive results, particularly in patients who are malnourished and with chronic inflammation [9]. Genetic testing for mutations and deletions in the GLA segment of the X chromosome is vital to obtaining the correct diagnosis in females and is required in males to rule out false-positive low alpha-galactosidase levels. Genetic testing is also necessary to assess if a mutation is amenable to oral chaperone therapy as an alternative to enzyme replacement therapy. This patient's genetic variation is an N228H mutation. We found this mutation responsive to migalastat based on (GLP-validated) HEK assay. Unfortunately, this mutation was not included in either the ATTRACT [8] or FACETS [10] studies of migalastat, so monitoring of serum GL-3 levels, kidney function, and left ventricular mass will be vital. Enzyme replacement is available for all patients with Fabry disease mutations but is required for patients with mutations that are not amenable to migalastat chaperone therapy.

The low incidence of diagnosing Fabry disease through screenings programs in patients at high risk such as ESRD, early-onset ischemic strokes, and left ventricular hypertrophy suggests that this form of screening may not be warranted. The incidence of Fabry disease in ESRD patients is upwards of 1% (one in a hundred). It suggests that nephrologists should not ignore the possibility of the disease and maintain a healthy index of suspicion.

More importantly, identifying Fabry disease in the ESRD population benefits the patient's family. A Spanish review of 3,650 dialysis patients identified only 11 new cases of Fabry disease but diagnosed 23 family members out of 66 tested [3]. A Turkish study of 1136 ESRD patients only found one new case of Fabry disease but identified six additional affected family members [4]. A French survey of 106 patients with ESRD found one new case of Fabry disease with seven new family members diagnosed [11]. Having a high index of suspicion for Fabry disease benefits both the patient and their family in avoiding the complications of this disease.

## Data Availability

The data are appropriately referenced in the article with citations that are listed at the end.

## References

[1] Linthorst G. E., Hollak C. E., Korevaar J. C., Van Manen J. G., Aerts J. M., Boeschoten E. W. (2003). Alpha-galactosidase A deficiency in Dutch patients on dialysis: a critical appraisal of screening for Fabry disease. *Nephrology Dialysis Transplantation*.

[2] Nakao S., Kodama C., Takenaka T. (2003). Fabry disease: detection of undiagnosed hemodialysis patients and identification of a “renal variant” phenotype. *Kidney International*.

[3] Herrera J., Miranda C. S. (2014). Prevalence of Fabry’s disease within hemodialysis patients in Spain. *Clinical Nephrology*.

[4] Okur I., Ezgu F., Biberoglu G. (2013). Screening for Fabry disease in patients undergoing dialysis for chronic renal failure in Turkey: identification of new case with a novel mutation. *Gene*.

[5] Rolfs A., Böttcher T., Zschiesche M. (2005). Prevalence of Fabry disease in patients with cryptogenic stroke: a prospective study. *The Lancet*.

[6] Kim W. S., Kim H. S., Shin J. (2019). Prevalence of Fabry disease in Korean men with left ventricular hypertrophy. *Journal of Korean Medical Science*.

[7] Tuttolomondo A., Simonetta I., Duro G. (2017). Inter-familial and intra-familial phenotypic variability in three Sicilian families with Anderson-Fabry disease. *Oncotarget*.

[8] Germain D. P., Hughes D. A., Nicholls K. (2016). Treatment of Fabry’s disease with the pharmacologic chaperone migalastat. *New England Journal of Medicine*.

[9] Jahan S., Sarathchandran S., Akthar S. (2020). Prevalence of Fabry disease in dialysis patients: western Australia Fabry disease study—the forward study. *Orphanet Journal of Rare Diseases*.

[10] Hughes D. A., Nicholls K., Shankar S. P. (2017). Oral pharmacological chaperone migalastat compared with enzyme replacement therapy in Fabry disease: 18-month results from the randomized phase III attract study. *Journal of Medical Genetics*.

[11] Bekri S., Enica A., Ghafari T. (2005). Fabry disease in patients with end-stage renal failure: the potential benefits of screening. *Nephron Clinical Practice*.

